# Durability Investigation of Ultra-Thin Polyurethane Wearing Course for Asphalt Pavement

**DOI:** 10.3390/ma17204977

**Published:** 2024-10-11

**Authors:** Wenguang Wang, Baodong Liu, Dongzhao Jin, Miao Yu, Junsen Zeng

**Affiliations:** 1 School of Civil Engineering, Beijing Jiaotong University, Beijing 100044, China; 23115146@bjtu.edu.cn (W.W.); bdliu@bjtu.edu.cn (B.L.); 2 Department of Civil, Environmental, and Geospatial Engineering, Michigan Technological University, 1400 Townsend Drive, Houghton, MI 49931-1295, USA; 3 School of Civil Engineering, Chongqing Jiaotong University, Chongqing 400074, China; yumiao@cqjtu.edu.cn (M.Y.); junsen3210@163.com (J.Z.)

**Keywords:** skid resistance, ultra-thin wear layer, durability

## Abstract

In this study, a wear-resistant ultra-thin wear layer was fabricated with polyurethane as an adhesive to investigate its durability for pavement applications. Its road performance was investigated based on indoor tests. First, the abrasion test was performed using a tire–pavement dynamic friction analyzer (TDFA), and the surface elevation information of the wear layer was obtained by laser profile scanning. The relationship between the anti-skid properties of the wear layer and the macro-texture was analyzed. Second, a Fourier infrared spectrometer and scanning electron microscope were employed to analyze the evolution of polyurethane aging properties in the pull-out test and accelerated ultraviolet (UV) aging test. The results showed that the mean profile depth (MPD), arithmetic mean wavelength of contour (λa), surface wear index (SBI), stage mass loss rate (σ), and total stage mass loss rate (ω) of the abrasive layer aggregate had significant multivariate quadratic polynomial relationships with the skidding performance of the abrasive layer. The tensile strength of the polyurethane ultra-thin abrasive layer decreased by only 2.59% after 16 days of UV aging, indicating a minimal effect of UV action on the aggregate and structural spalling of the polyurethane abrasive layer.

## 1. Introduction

Asphalt pavements are prone to cracking, loosening, congestion, and deformation under the long-term joint action of load and environment [1,2,3,4,5], weakening the anti-skid performance and reducing the service life of the pavement [4,6,7,8]. Currently, the common measures to prevent pavement distress and restore the skid resistance of pavement include micro-surfacing, gravel sealing, thin-slurry sealing, and various ultra-thin wear layers [9,10,11]. Compared with the first three technologies, among these, ultra-thin wear layers, particularly those using advanced polymer materials, have gained attention due to their superior durability, cost-effectiveness, and enhanced skid resistance [12,13,14,15,16,17,18]. For example, Novachip has low noise, favorable abrasive resistance, and anti-skid performance [19]. With the emergence of various polymer materials, there are more ways to apply ultra-thin abrasive layers, which have smaller thicknesses than traditional abrasive layers [2,20,21]. Cuadri et al. [22,23] used castor oil prepolymer as a modifier to prepare 90 °C modified asphalt. At the same temperature, the viscosity of polyurethane-modified asphalt with a 2% admixture was higher than the SBS-modified asphalt with a 3% admixture, suggesting an improved high-temperature performance. Li et al. [24] investigated the bonding of asphalt to an aggregate in permeable asphalt concrete (AC) pavements and ultra-thin overlays. Through laboratory tests, they have successfully developed a high-viscosity liquid asphalt that requires no preheating and can be applied directly at room temperature with excellent performance after curing. However, due to the low strength, easy aging, and poor high-temperature stability of the asphalt, the asphalt ultra-thin abrasive layer is prone to insufficient skid resistance. Similarly, the epoxy resin material suffers from easy aging and poor weathering resistance, resulting in cracking and interlayer spalling of the wear layer.

Compared with asphalt and epoxy resin, polyurethane material has better toughness, strength, weather resistance, temperature stability, and corrosion resistance [25,26,27,28,29]. Lu et al. [30] found that the ultra-thin polyurethane wear layer had excellent resistance to abrasion and aging after replacing asphalt with a polyurethane binder. In his study, the curing process of polyurethane/epoxy-modified asphalt was examined, with the results showing that an interpenetrating network structure formed, improving mechanical strength and flexibility [31]. Liao et al. investigated the shear bond strength of porous elastic pavement to the subgrade and developed polyurethane-cemented granules as a binder [32]. Hong et al. proposed the polyurethane ultra-thin friction layer as a new tunnel pavement material. The results showed that increasing the isocyanate index could significantly improve the mechanical properties and water resistance of polyurethane materials. Wang et al. conducted an in-depth study on the frictional properties of polyurethane ultra-thin wear layers. The friction properties and wear resistances of the materials were analyzed by friction coefficient tests and friction wear experiments. The results showed that the polyurethane ultra-thin wear-resistant layer had a high coefficient of friction and good abrasion resistance, which can provide good anti-skid performance for the pavement [33]. Deng et al. investigated the correlation between ultra-thin wear layer aggregates and skid resistance through a molecular dynamics (MD) simulation and gray correlation methods [34]. Hong et al. studied the new polyurethane ultra-thin wear layer for tunnels and revealed its excellent skid resistance, fire-retardant properties, and environmental friendliness, with great potential in the future construction of tunnel pavements [35].

As mentioned above, traditional wear-resistant layers tend to suffer from a rapid decline in anti-skid performance and poor durability during service [17,18,36,37,38]. However, previous studies have mainly focused on material performance and functionality, with less focus on the anti-skid and anti-peeling properties of polyurethane ultra-thin wear layers. In addition, the skid and abrasion resistance of the pavement wear layer is related to both the characteristics of the binder and the bonding effect between layers and the molding method of the wear layer [39]. Therefore, its performance degradation under different external conditions needs to be further investigated. This study aims to address the gaps identified in previous studies and provide a comprehensive evaluation of polyurethane wear layers for asphalt pavements

The objective of this study was to evaluate the road durability of polyurethane ultra-thin wear layers by investigating their anti-skid performances and resistance to delamination. Using a dynamic tire–pavement tribometer, the frictional effects of tire interaction on the wear layer were simulated, with three-dimensional surface profile data collected via laser scanning before and after testing. The study analyzed the evolution of surface macro-texture and its impact on skid resistance, as well as the attenuation of anti-skid performance over time. In addition, shear strength and tensile strength were measured to assess the interlayer bonding performance, while the effects of ultraviolet (UV) aging on the surface microstructure and wear layer delamination were examined through scanning electron microscopy (SEM) and infrared spectroscopy.

## 2. Materials and Methods

### 2.1. Raw Materials

#### 2.1.1. Polyurethane Adhesives

The general physical and chemical properties were tested according to JT/T712-2008 [40], GB/T1720-2020 [41], and GB/T 3186-2006 [42] specifications. The test results are shown in Table 1.

#### 2.1.2. Abrasion-Resistant Aggregates

The wear-resistant aggregates utilized in the experimental procedure included basalt, ceramic particles, adamantine, and manufactured sand. The main technologies of the aggregates were tested according to JTG E42-2005 specifications [44], and the results are shown in Table 2.

#### 2.1.3. Preparation of Polyurethane Ultra-Thin Wear Layer

The AC-13 slab specimens were prepared according to the specification requirements [45]. First, the surface layer was processed by cleaning the surface of the 300 mm × 300 mm × 30 mm slab. Second, components A and B of the polyurethane material were mixed evenly according to the ratio of 4:1 [46,47]. A total of 100 g mixture was weighed and evenly applied on the surface of the slab. Finally, the aggregate was spread evenly and left to cure and mold naturally before removing the excess aggregates.

### 2.2. Anti-Skid Performance Test

Tire–pavement dynamic friction analyzer (self-invented) and laser profile scanners (MTI Instruments, Inc., model LTS-050-20, Albany, NY, USA) are the main instruments for skid-resistance testing. The TDFA system can obtain the dynamic friction coefficient (DFC) between the tire tracks under different working conditions in real time. The laser profile scanner acquires the 3D profile data of the wear layer surface after different wear times, allowing for a comparative analysis of the macroscopic texture parameters of the wear layer surface under different test times.

#### 2.2.1. Laser Profile Scanning Test

(1) Data collection method

**Figure 1:**
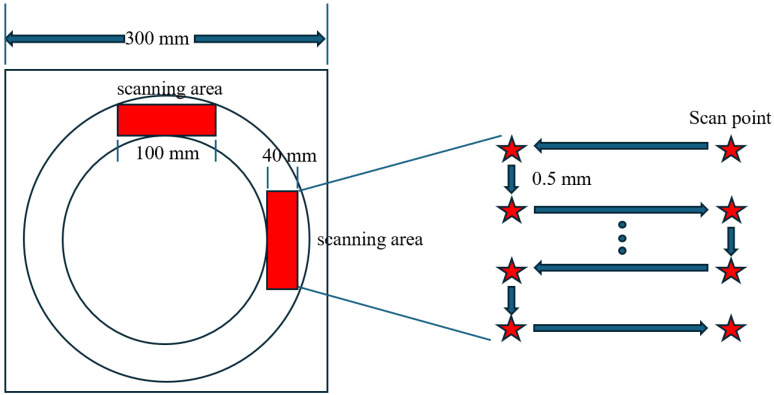
Macro texture measurement area and scan path.

Macrotexture data were collected at various process stages using a laser profile scanner. Specifically, scanning tests were performed at 0, 0.5, 1, 2, 4, 6, 8, 10, 12, and 14 h after wear. The scanning area consisted of two rectangular sections perpendicular to the wheel track belt, each measuring 40 mm × 100 mm. The scan path was S-shaped, and the data were collected every 0.03 s. The height test range was set to ±12 mm, and the vertical displacement was controlled at 0.5 mm, as shown in Figure 1.

(2) Texture parameter index selection

**Figure 2:**
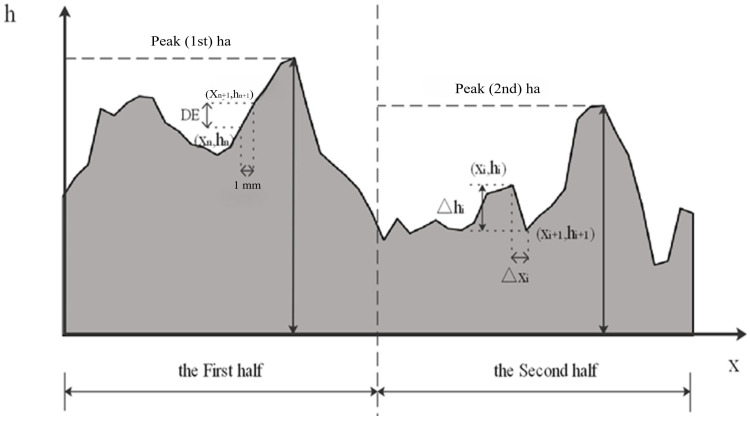
Schematic diagram of surface texture parameters.

Four types of parameter indexes were selected, including the amplitude parameter, spacing feature, shape feature, and integrated feature. The relevant formulas are shown in Table 3, and the semantic parameter annotations are shown in Figure 2.

#### 2.2.2. DFC Test

DFC measurements were alternated with laser profile scanning tests. The ratio of polyurethane host agent to vulcanizing agent was 4:1, and the dosage was 1.0 kg/m^2^.

(1) DFC testing under different loads

Wear layers prepared by 2~4 mm basalt aggregates were tested at a speed of 30 km/h, and the test loads were 250 N, 350 N, and 450 N.

(2) DFC test at different speeds

Wear layers prepared from 2 to 4 mm basalt aggregate were tested at 20 km/h, 30 km/h, and 40 km/h, with a test load of 250 N.

(3) DFC testing with different aggregate types

Wear layers were prepared using basalt, ceramic, and adamantine, with particle sizes of 2–4 mm. The test speed was 30 km/h, and the test load was 250 N.

(4) DFC test with different aggregate particle sizes

The basalt wear layer was prepared with aggregates of 1–2 mm and 2–4 mm, with a TDFA test speed of 30 km/h and a test load of 250 N.

### 2.3. Anti-Peeling Performance Test

#### 2.3.1. Bonding Performance Test

(1) Tensile strength test

The pull-out specimens [48] were prepared with reference to ASTM C1583/C1583M-20 [49], and the diameter of the pull-out head was 50 mm. The asphalt mixture specimens (I) and abrasive layer specimens (II) were tested using 1.0 kg/m^2^ of polyurethane adhesive at 15 °C, 25 °C, 35 °C, and 50 °C. Three samples of each group were prepared and left for 24 h, and the average values were taken.

The pull-out strength can be calculated by the following Equation (1):

(1) $$\sigma =\frac{F}{\pi {\left(\frac{D}{2}\right)}^{2}}$$

where *σ* is the pulling strength (MPa); *F* is the maximum pulling force displayed by the pulling instrument (N); and *D* is the diameter of the disc at the bottom of the pulling head (mm).

**Figure 3:**
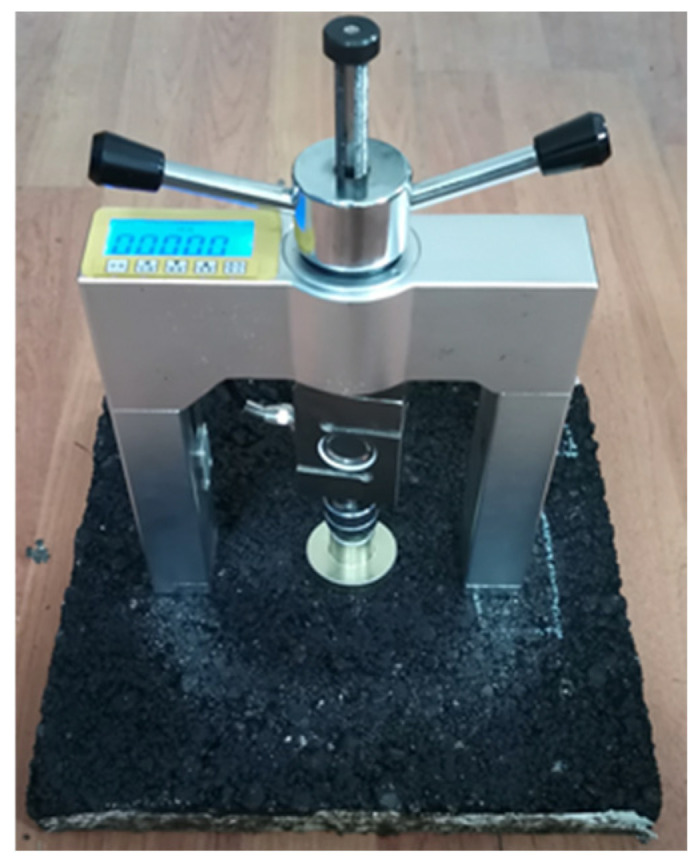
Physical diagram of the pull-out test.

The physical diagram of the pull-out test used in this study is shown in Figure 3. This test determines the tensile strength of asphalt mixture specimens and abrasive layer specimens [20].

(2) Shear-strength test

**Figure 4:**
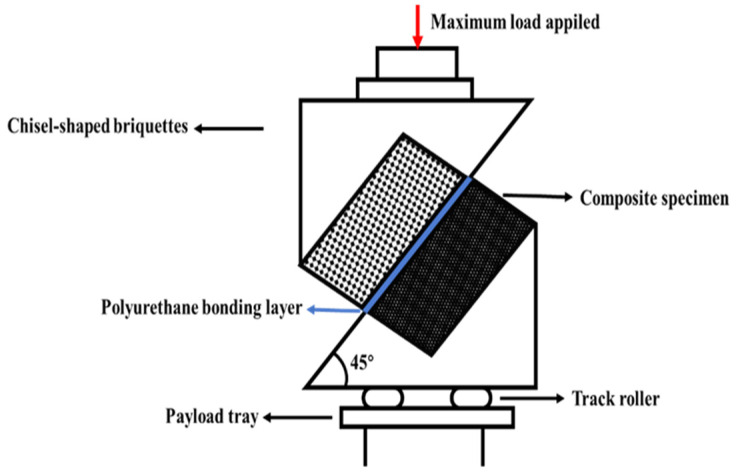
Shear test force diagram.

In this study, a 45° oblique shear test was conducted to assess the shear strength between the wear layer and the underlying pavement. The Shear test force diagram is shown in Figure 4. To simulate the shear forces exerted by vehicle tires, composite specimens were prepared with dimensions of 100 mm × 50 mm × 100 mm. The test was performed using a 100 kN universal testing machine (source: UTM100, IPC Global, Melbourne, Australia) set to a displacement rate of 50 mm/min. This configuration was designed to accurately represent the real-world shear stresses applied to the wear layer during vehicle operation, ensuring a reliable evaluation of its bonding strength and durability under simulated conditions. The number of effective parallel specimens was 3, and the test results were averaged.

The shear strength between the polyurethane ultra-thin wear layer and the original pavement layer can be calculated by Equation (2).

(2) $$\tau =\frac{F\times \sin\alpha }{S}$$

where *τ* is the shear strength (MPa), *F* is the maximum load applied (N), *S* is the cross-sectional area of the specimen (mm^2^), and *α* is the angle of force on the specimen (45°).

#### 2.3.2. Anti-UV Aging Performance Test

An indoor accelerated UV aging test was conducted to simulate the effects of UV light on a polyurethane ultra-thin wear layer in the Sichuan basin, China. The annual solar radiation in this area is 4150 MJ/m^2^, of which 249 MJ/m^2^ is UV radiation. The calculated time for two years of simulated natural UV aging with indoor acceleration was 1.36 × 10^6^ s, for a total of 378.8 h (about 16 days).

The dumbbell specimen and the wear layer specimen were used in the test. The aging times for the dumbbell specimen were set at 0, 2, 4, 6, 9, 12, and 16 days.

(1) SEM microscopic observation

Tensile specimens of polyurethane materials that were not aged, aged for 4 days, and aged for 16 days were selected for SEM observation.

(2) Fourier infrared spectroscopy test

In order to explore the UV aging mechanism of polyurethane materials, the FTIRs of the specimen surfaces after different days of aging were tested separately.

(3) Tensile performance test

The evolution of the tensile properties of polyurethane specimens after different aging days was tested using an universal testing machine, referred to as ASTM D638-2014 [50]. The tensile speed was 5 mm/min. The effective number of parallel specimens was 4, and the test results were averaged.

(4) Anti-peeling performance test

The spalling of the aggregates from the polyurethane ultra-thin wear layers after aging treatment was analyzed. Specimen A was the initial unworn specimen after molding, and specimen B was the specimen after 14 h of abrasion. Both specimens were placed into the UV aging for 16 days of accelerated aging before being taken out. Subsequently, Specimen A was abraded for 14 h, and Specimen B was abraded for 3 h to compare and analyze the spalling of the aggregate.

(5) Pulling performance test

With the configured polyurethane mastic, the pull-out head is bonded to the rutted plate in the aging wear layer, which is left to stand for 1 day to carry out the pull-out test and determine the pull-out strength of the aging specimen.

**Figure 5:**
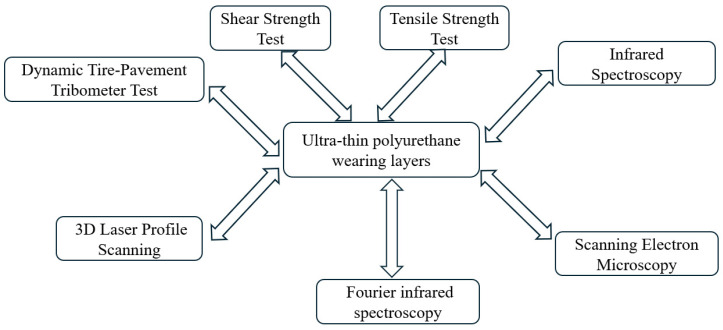
A flowchart of test program in this study.

The experimental procedure for this study is outlined in Figure 5. Laboratory-prepared ultra-thin polyurethane wear layers will undergo various tests, including the dynamic tire–pavement tribometer test, 3D laser profile scanning, shear-strength test, tensile strength test, infrared spectroscopy, scanning electron microscopy (SEM), and Fourier infrared spectroscopy. These tests will provide comprehensive data on the performance and durability of the polyurethane layers.

## 3. Results and Analysis

### 3.1. Wear-Resistant Performance Analysis

#### 3.1.1. Texture Parameter Evolution Analysis

In contrast to the other three aggregates, manufactured sand is no longer included in comparative analyses of subsequent aggregates, given the clear evidence of abrasion in the ultra-thin layer. Moreover, the basalt wear layer is adopted to evaluate the effect of the evolution of different texture parameters on the anti-skid performance through correlation analysis. Table 4 presents the coefficients of determination (R^2^) between various texture parameters and the dynamic friction coefficient, among others. The R^2^ value indicates the proportion of variance in one variable that is predictable from another variable, providing insights into the strength of their relationship.

When the coefficient of determination R^2^ between the two parameters is less than 0.6 [51], the two parameters describe different texture features. As can be seen from Table 4, there are significant correlations between MPD and Ra, Rq, and Δq (R^2^ > 0.8) and strong correlations between λa, λq, and MDE (R^2^ > 0.6), indicating a similar characterization of the wear layer texture features by the above parameters. Therefore, only the most representative parameter, MPD, was selected, and the pavement friction coefficient regression model was constructed with the introduction of λ_a_, R_sk_, K_u_, and SBI.

**Figure 6:**
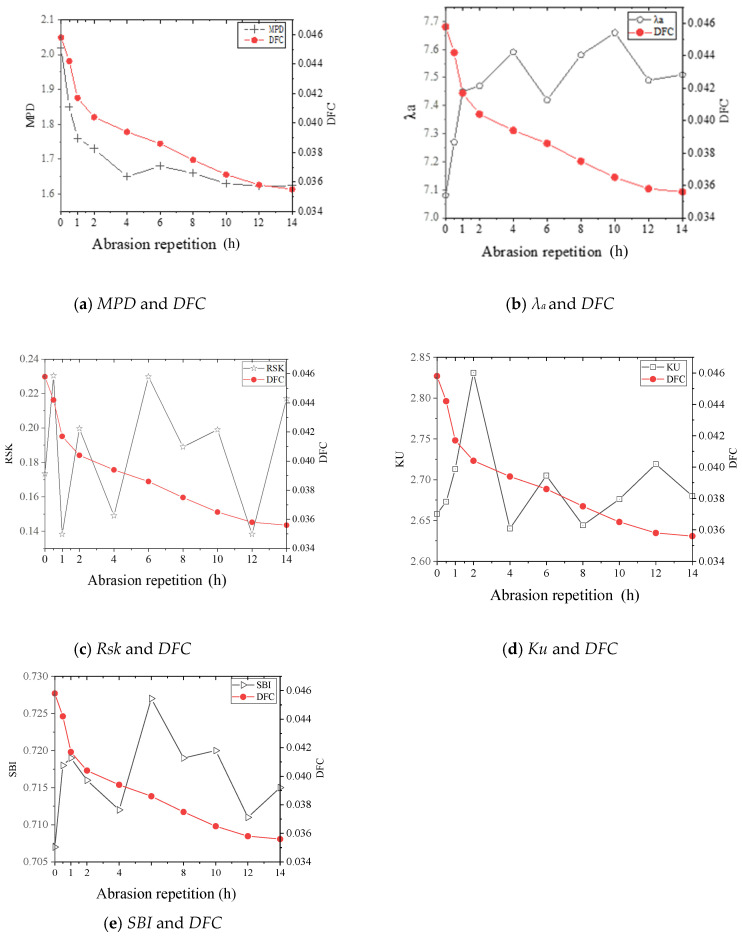
Variation rule of each texture parameter and DFC with abrasion time.

As shown in Figure 6, among the five parameters of λa, Rsk, Ku, SBI, and MPD, there is no simple linear relationship between them, except for the obvious linear correlation between MPD and DFC. Therefore, the five texture parameters can be adopted to establish the skid-resistance decay model.

#### 3.1.2. Decay Law of DFC

**Figure 7:**
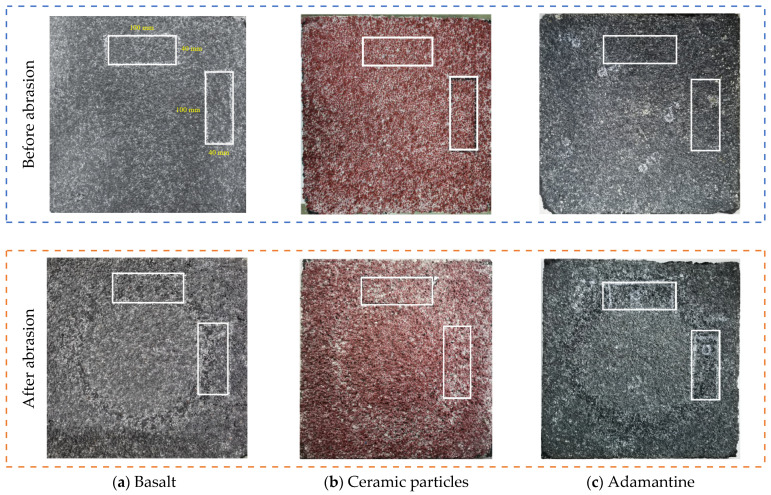
Morphology of three wear layers before and after abrasion. Note: all the scanned areas are two mutually perpendicular 40 mm × 100 mm rectangular areas on the wheel track.

The morphological changes of rutted plates before and after wear with three different types of aggregates are shown in Figure 7. The scanned area is two mutually perpendicular 40 mm × 100 mm rectangular areas on the wheel track.

(1) Effect of load on DFC decay

Basalt with a particle size of 2–4 mm was used to formulate 1.0 kg/m^2^ of polyurethane binder for the abrasion test of the specimens. The test speed was 30 km/h, and the test loads were 250 N, 350 N, and 450 N. The DFC changes are shown in Figure 7.

**Figure 8:**
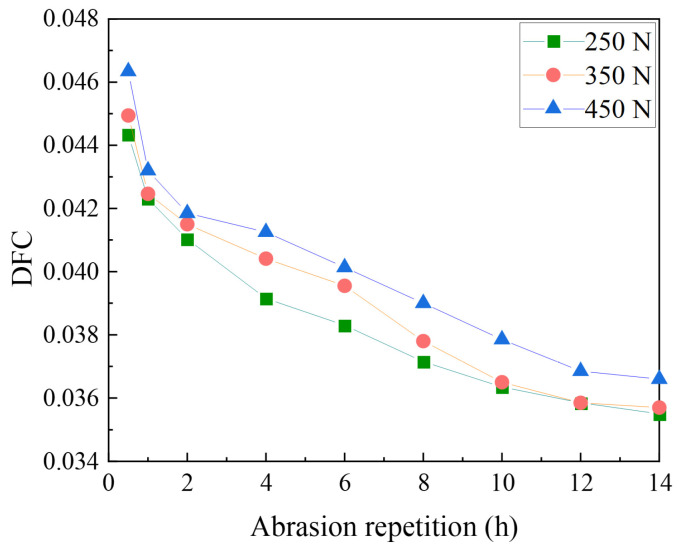
Decay of pavement DFC under different loads.

As shown in Figure 8, the initial values of DFC are 0.044, 0.045, and 0.046 when the loads are 250 N, 350 N, and 450 N, respectively. After 14 h of wear, the DFC under the load of 450 N is the highest, and the DFC under 250 N is the lowest. With higher loads, the effective pressure of the tire on the wear layer surface is greater, increasing the frictional resistance of the tire.

(2) Effect of velocity on DFC decay

**Figure 9:**
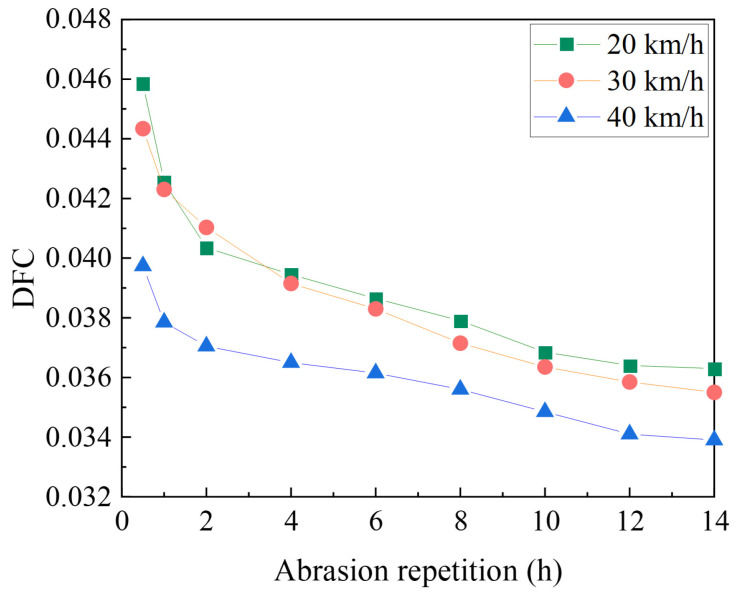
Decay of DFC at different velocities.

A polyurethane binder of 1.0 kg/m^2^ was prepared using basalt with a particle size of 2–4 mm for the abrasion test of the specimens. The test speeds were 20 km/h, 30 km/h, and 40 km/h, and the test load was 250 N. The variation in the DFC is shown in Figure 9.

As shown in Figure 9, the initial values of DFC are 0.046, 0.044, and 0.040 at vehicle speeds of 20 km/h, 30 km/h, and 40 km/h, respectively. At the same load, the effective contact area between the tire and the wear ply becomes smaller as the vehicle speed increases. As a result, the frictional resistance suffered by the tire is smaller, lowering the measured value of DFC.

(3) Effect of Aggregate Type on DFC Decay

**Figure 10:**
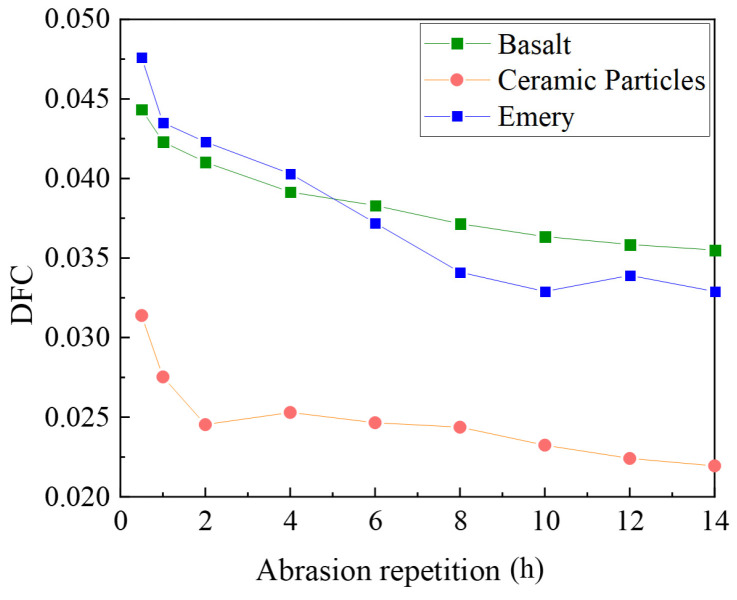
DFC decay of pavement with different aggregate types.

Basalt, ceramic, and adamantine, with a particle size of 2–4 mm, were used to prepare 1.0 kg/m^2^ of polyurethane binder. The molded specimens were used for the abrasion test at a speed of 30 km/h and a load of 250 N. The variation in the DFC is shown in Figure 10.

As shown in Figure 9, the dynamic DFC initial values of basalt, ceramic particles, and diamond abrasive layer are 0.044, 0.031, and 0.048, respectively. Ceramic particles have the smoothest surface, and their prepared abrasive layer has the smallest frictional resistance to the tire during friction with the tire. The diamond surface is the roughest, followed by basalt, which is prepared with similar initial and final values of DFC for the abrasive layer.

(4) Effect of aggregate particle size on DFC decay

**Figure 11:**
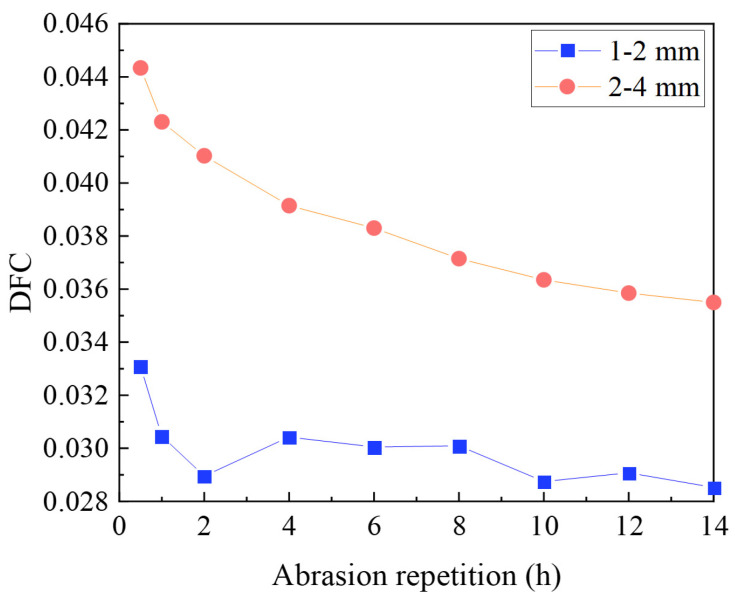
Decay of DFC with different aggregate particle sizes.

Basalt with particle sizes of 1–2 mm and 2–4 mm was used as wear-resistant aggregate and 1.0 kg/m^2^ of polyurethane cement was prepared. Wear tests were conducted on molded basalt specimens at a test speed of 30 km/h and a test load of 250 N. The changes in the DFC during the whole test are shown in Figure 11.

The DFC reduction in the abrasive layer with 1–2 mm and 2–4 mm particle size is 13.9% and 19.9%, respectively. With the same amount of binder, the small and medium grain-sized aggregates in the wear layer can be better encapsulated, losing the surface macrostructure and reducing the skid resistance.

(5) Aggregate quality loss analysis of the wear layer

**Figure 12:**
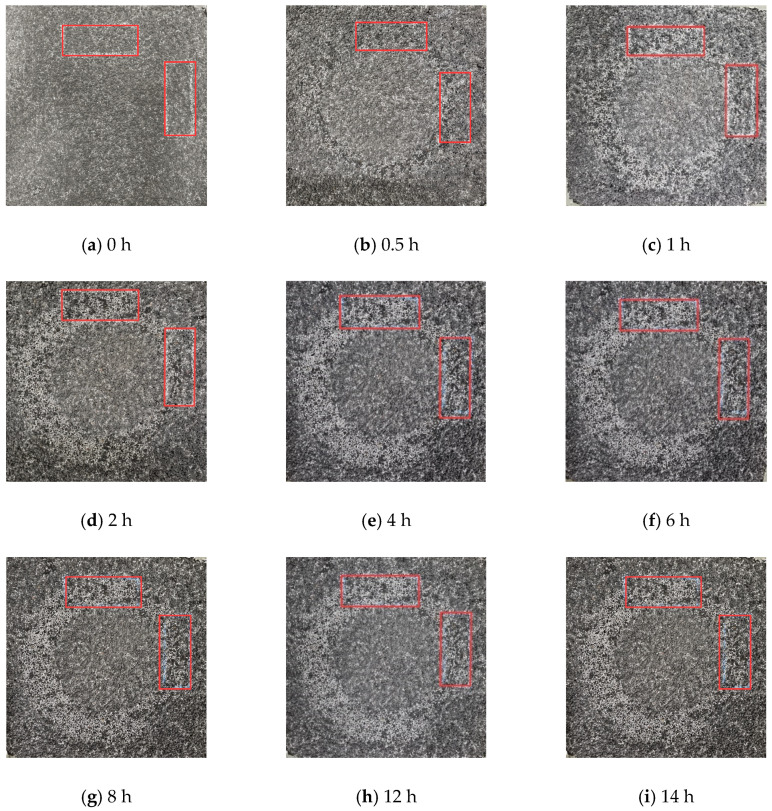
Wear layer appearance after different abrasion times. Note: all the scanned areas are two mutually perpendicular 40 mm × 100 mm rectangular areas on the wheel track.

There are differences in the mass loss rate of the aggregate in the abrasive layer under different single-factor conditions. The total mass loss rate (ω) and stage mass loss rate (σ) of aggregates were analyzed with speed as a variable. The results are shown in Table 5 and Figure 12.

(3) $$\omega =\frac{m_{0}-m_{1}}{m_{2}}$$

(4) $$\sigma =\frac{m_{i}-m_{i+1}}{m_{2}}$$

where *ω*—the total quality loss rate, %;

*m*_0_—the specimen quality without wear and tear, g;

*m*_1_—the quality of specimens after abrasion at different abrasion times, g;

*m_i_*, *m_i_*_+1_—the masses of the specimens before and after the adjacent abrasion time, respectively, g;

*m*_2_—the wear layer’s original quality, g.

**Table 5:** Aggregate mass loss rate under velocity variables.

Abrasion Time/h	20 km/h, 250 N		30 km/h, 250 N		40 km/h, 250 N	
	*ω*/%	*σ*/%	*ω*/%	*σ*/%	*ω*/%	*σ*/%
0.5	-					
1	2.68	0.93	2.92	1.01	3.78	1.55
2	3.37	0.7	3.67	0.75	4.79	1.01
4	3.73	0.36	4.23	0.56	5.32	0.53
6	4.08	0.34	4.80	0.56	5.86	0.54
8	4.38	0.31	5.00	0.20	6.18	0.33
10	4.59	0.21	5.21	0.20	6.56	0.38
12	4.8	0.21	5.35	0.15	6.73	0.17
14	4.96	0.15	5.48	0.13	6.85	0.12

Table 5 shows that the relationship between the size of the aggregate mass loss rate under the action of three speeds is 40 km/h > 30 km/h > 20 km/h under the same wear time. The analysis shows that the faster the speed, the more times the same location is subjected to the action per unit of time, and the greater the probability of aggregate spalling.

#### 3.1.3. Skid Resistance and Its Decay Analysis

(1) Analysis of the anti-skid decay process

From the above study, it can be seen that the decay of the DFC of a polyurethane ultra-thin wear layer under different working conditions has some differences, but the overall decay curve can be roughly divided into a fast decay zone, a slow decay zone, and a decay stability zone.

The rapid decay stage is dominated by the continuous spalling of loose aggregate on the surface of the wear-resistant layer. The friction force on the loose aggregate is greater than its bonding force with the binder. The aggregate will spall off one after another, and the DFC decreases faster. The slow decay stage is mainly dominated by the wear and abrasion of the aggregate surface. The results indicate that the loose aggregate on the surface of the wear layer has been basically stripped. When the aggregate stripping and abrasion reach a certain degree, the skid resistance of the abrasive layer decays into a stable stage.

(2) Anti-skid decay model based on texture evolution of the road surface

In this paper, the DFC, with an operating speed of 30 km/h measured by a TDFA, was used as the research object, and 250 sets of asphalt pavement texture-characterization data and dynamic friction skid-resistance data were collected to establish the skid-resistance attenuation model based on the evolution of the pavement texture. A multivariate linear stepwise regression analysis was conducted with five parameters, namely MPD, λa, Rsk, Ku, and SBI, as independent variables and with DFC as a dependent variable. The coefficient of determination R^2^ of the optimal regression equation is 0.708, but only the MPD is utilized. No other parameters are not involved. Therefore, the regression of the multivariate quadratic polynomial model will be performed using SAS analysis software.

For subsequent analysis, the five selected independent variables are combined into a synthetic matrix, denoted as M = [MPD λa Rsk Ku SBI], and the expression for the multiple quadratic polynomial regression model can be set as Equation (5).

DFC = MAM T + BMT + C(5)

where DFC is the dynamic friction coefficient, A is the quadratic term coefficient matrix, B is the primary term coefficient matrix, C is the constant term, and T is the matrix transpose.

The model coefficient of determination R^2^ of 0.82 was obtained by multivariate quadratic polynomial regression with five parameters, including MPD, and the results of the factor significance test are shown in Table 6.

Table 6 shows that the probability P of the F-test of the five independent variables is greater than the significance level of 0.05, indicating that the five parameters mentioned above have poor significance levels as model parameters for factorial regression. There are still non-significant terms and non-significant factors in the regression model, indicating that the decay of the anti-skid performance of the polyurethane wear layer cannot be reasonably described by only using the road surface texture as the indicator.

Aggregate spalling can affect abrasive layer pavement texture. The mass loss rate (σ) of the aggregate stage of the wear layer and the total mass loss rate (ω) of the aggregate stage during the test will be introduced as new independent variables, denoted as M1 = [X1 X2 X3 σ ω], where X1, X2, and X3 are taken from M, and the terms in M can be crossed and combined. After several binomial fitting analyses, all aspects of the model are satisfied when M1 = [MPD λa SBI σ ω], and the parameters A, B, and C are shown in Equation (6).

(6) $$A\;=\left[\begin{array}{ccccc} -0.1183 & -0.0444 & -0.1853 & -0.0104 & 0.0113\\ -0.0444 & -0.0021 & 0.0001 & 0.0018 & 0.0115\\ -0.1853 & \;0.0001 & -0.5621 & 0.0299 & 0.0763\\ -0.0104 & 0.0018 & \;0.0299 & 0.0000 & \;0.0005\\ 0.0113 & 0.0115 & \;0.0763 & 0.0005 & \;0.0019 \end{array}\right];\;B\;=\left[0.9096\;0.0884\;0.9195-0.0194-0.1571\right];\;\;C\;=-1.2807\;\;\;$$

As shown in Table 7, the coefficient of determination R^2^ of the model is 0.9890, the *p*-values of the linear term, the quadratic term, and the total model F-test are all *p* < 0.0001, the *p*-value of the interaction term is 0.0488, and the *p*-values of all four terms are below the significant level of 0.05. The results indicate that there is a significant quadratic polynomial relationship between the explanatory variables and the explained variables in the model. To further confirm the significance of the model, each of the five factors of the model was tested and analyzed. Table 8 shows that the *p*-values of the five-factor F-tests for MPD, λa, SBI, σ, and ω are less than the significance level of 0.05. The results indicate that the regression coefficients of the factors are significant. The SAS regression results indicate that the attenuation of the texture parameter of the road surface alone does not well-characterize the decay of the skid resistance of the polyurethane wear layer. Furthermore, the aggregate stage mass loss rate (σ) and total stage mass loss rate (ω) were introduced as factors to form a synthetic matrix M1 with MPD, λa, and SBI. The coefficients of determination and the significance of factors of the fitted quadratic polynomial model meet the requirements, indicating that the relevant indexes can accurately describe the decay of the skid resistance of the abrasive layer.

### 3.2. Anti-Spalling Performance Analysis

#### 3.2.1. Interlayer Bond Strength of the Abrasive Layer

(1) Tensile strength

**Figure 13:**
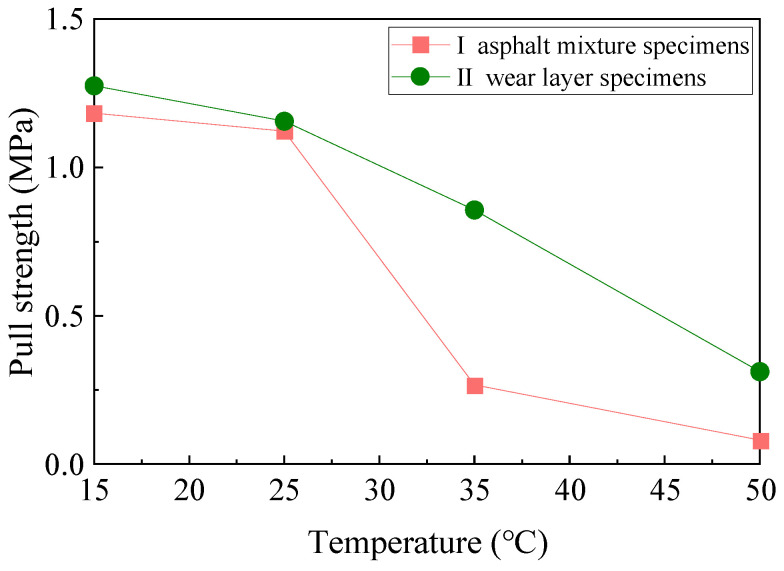
Variation of drawing strength of two specimens with temperature.

The trends in the pull-out strength of the asphalt mixture specimens (I) and wear layer specimens (II) at different temperatures are shown in Figure 13.

As shown in Figure 13, the tensile strength of both the asphalt mixture specimens and the wearing course specimens decreases as the temperature increases. At higher pavement temperatures, the bond strength within the asphalt mixture decreases due to the softening of the asphalt. Meanwhile, polyurethane is a thermosetting material with good high-temperature stability. The pull-out strengths of the abrasive layer specimen at 35 °C and 50 °C are much higher than that of the asphalt mixture specimen, indicating that the polyurethane abrasive material can inhibit the high-temperature damage of asphalt pavement. The damage location of both specimens is not inside the asphalt mixture when the temperature is low, indicating that both asphalt and polyurethane have good low-temperature performance. When the temperature gradually increases, the performance of asphalt gradually decreases. And the adhesion inside the asphalt mixture decreases, while the polyurethane material still has good adhesion strength with the original pavement.

(2) Shear strength

Table 9 shows that the shear coefficients of variation of the composite specimens at the three temperatures are 1.16%, 2.85%, and 7.49%, respectively, which are less than 10%. The results indicate that the test data are more reliable. The average shear strengths of the specimens are 3.58 MPa, 1.57 MPa, and 0.59 MPa, respectively, with −228.0% of the shear strength at 15 °C and 37.6% of the shear strength at 50 °C for 25 °C. The shear stress-displacement curves of the specimens under high-temperature conditions are different from the other two cases. The displacement curve did not show a rapid decrease in shear after reaching the maximum value but continued to decrease slowly. The results occur because asphalt is a thermoplastic material, while polyurethane is a thermosetting material.

#### 3.2.2. Effect of UV Aging on the Peeling Properties of Abrasive Layers

(1) SEM observation results

**Figure 14:**
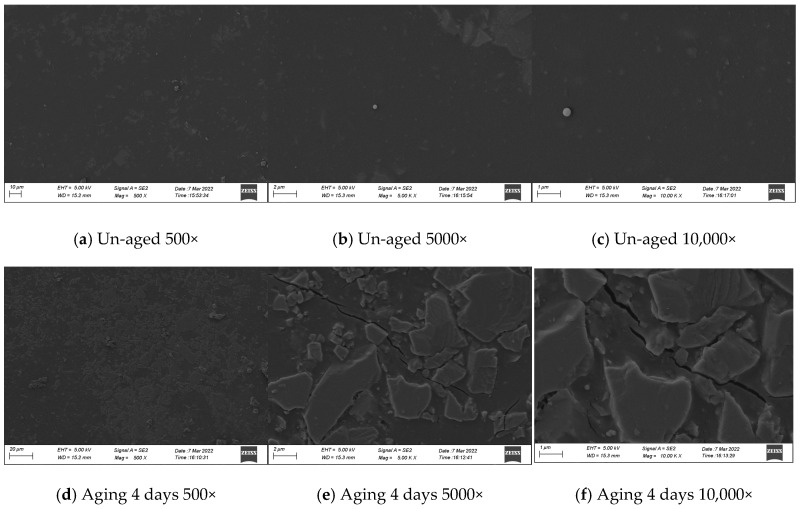

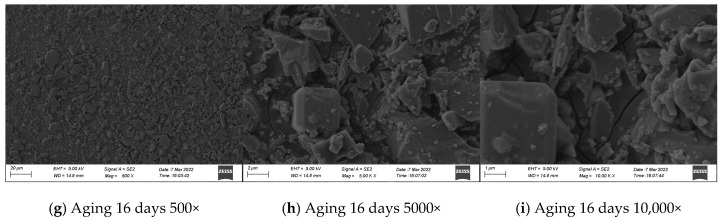
Changes in the apparent morphology of polyurethane specimens after UV aging.

As shown in Figure 14, the surface of the unaged specimen is flat and crack-free. After aging for four days, irregularly shaped patches and interlaced cracks appeared on the surface of the specimen. After aging for 16 days, the surface of the specimen gradually increased in size and became more three-dimensional, and cracks increased. It can be inferred from the results that the main reason for the appearance of irregular patches and cracks on the surface of the specimen is the change in the chemical structure of polyurethane.

(2) Fourier infrared spectroscopy test

**Figure 15:**
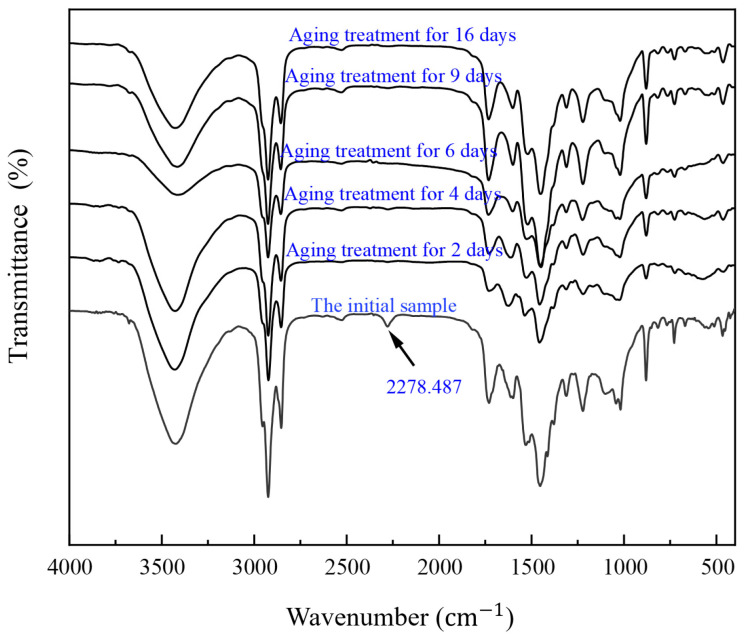
Infrared spectral evolution of polyurethane material after UV aging.

The infrared spectra of the initial specimens of polyurethane materials were similar to those in the study [52,53,54,55]. As shown in Figure 15, 3424 cm^−1^ is the peak associated with carbamate, and the overall peak area at this position shows a decreasing trend, indicating that the structure of the polyurethane hardness part has been damaged. Furthermore, 2923 cm^−1^ and 2853 cm^−1^ are the stretching vibration peaks of methylene (CH_2_), and the reason for the change in the absorption peak is that the methylene is oxidized. Initially, there is a clear absorption peak and a stretching vibration of -N=C=O at this location at 2278 cm^−1^, and the unreacted -N=C=O functional group in the specimen completely disappears after two days of UV aging. The intensity of the absorption peak at 1730 cm^−1^ decreases first and then increases with aging time. It is speculated that the reason is that the C=O bond breaks at the initial aging stage and forms a new group structure.

(3) Tensile properties

**Figure 16:**
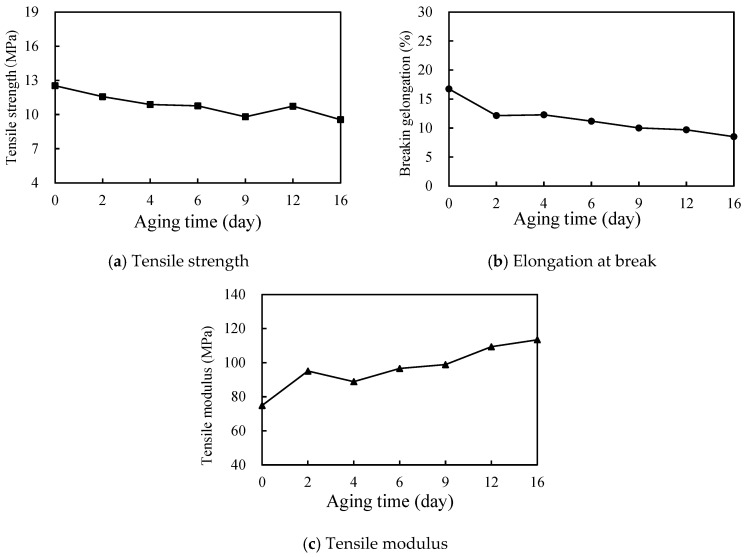
Law of the effect of UV aging on the tensile properties of polyurethane.

Figure 16 shows that the tensile strength of the polyurethane specimens shows a certain degree of decay in general with the aging time. However, after aging for 12 days, the tensile strength shows a rebound phenomenon and recovers to 85.6% of the initial strength. At 16 days of aging, the tensile strength is 76.2% of the initial strength, for a decrease of 23.8%.

As shown in Figure 16b, UV aging harms the elongation at the break of polyurethane materials. At two days of aging, the elongation at the break shows a large decrease of 27.4%. At 16 days of aging, the elongation at the break is 50.9% of the initial state, for a decrease of 49.2%. The results show that UV aging decreases the elongation at the break of polyurethane materials.

Figure 16c shows that the tensile modulus of the polyurethane specimens increased by 27.0% within two days of aging. The tensile modulus decreases at 4 days of aging and is similar to the modulus at 6 days of aging. After 16 days of aging, the modulus increases to 151.6% of the initial value, for an increase of 51.6%. The results show that, instead of decreasing the tensile modulus of polyurethane materials, UV aging promotes the increase in tensile modulus, which leads to the hardening of the materials after aging.

In summary, after two years of simulated UV aging (16 days of accelerated aging), the tensile strength and elongation at the break of polyurethane materials decrease to a certain extent while the tensile modulus increases, and the increase is large. The reason for the results is that the molecular structure of the C-N bond and C-O bond in polyurethane specimens will gradually break during aging, thus reducing the tensile properties and accelerating the destruction of the overall structure of the polyurethane ultra-thin wear-resistant layer to a certain extent.

(4) Anti-spalling performance analysis

**Figure 17:**
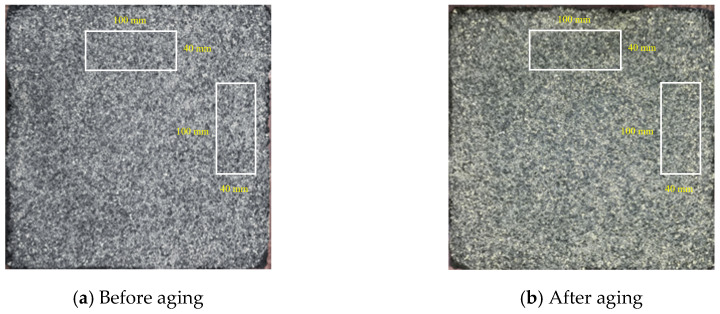
The appearance of A Type wear layer before and after aging.

**Figure 18:**
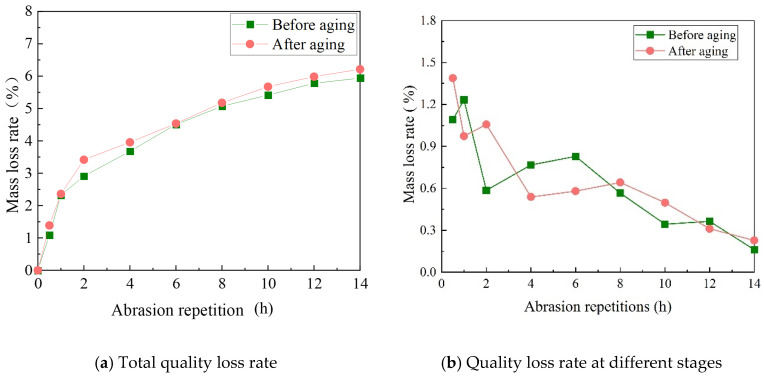
Mass loss of class A abrasive layer before and after UV aging.

Figure 17 illustrates the visual changes in the appearance of Type A polyurethane wear layer specimens before and after UV aging. In contrast, Figure 18 presents the stage mass loss rate and total mass loss rate of both the unaged diamond abrasive and the Type A aged abrasive layers. After 16 days of accelerated UV aging indoors, the surface aggregate spalling of the polyurethane ultra-thin wear layer showed minimal differences compared to the unaged wear layer with the same aggregate type. The exponential trend lines for the aggregate mass loss rates at various wear stages are nearly identical, indicating that UV aging had a limited effect on the wear resistance of the polyurethane layer. To enhance clarity, the scale has been added in Figure 17.

**Figure 19:**
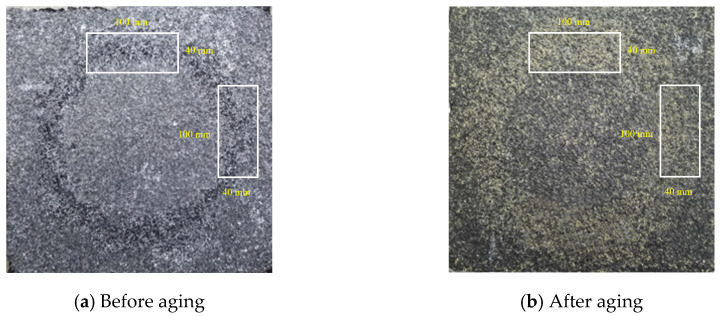
Class B wear layer specimens before and after aging.

**Figure 20:**
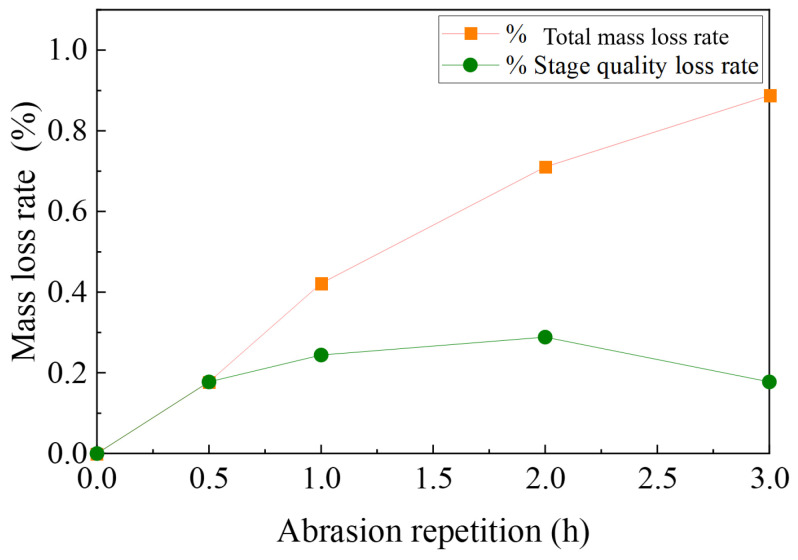
Mass loss of class B wear layer after UV aging.

Figure 19 shows the morphological changes in the appearance of the polyurethane wear layer before and after UV aging of the Type B wear layer. Figure 20 shows the mass loss rate of this abrasive layer after 3h of continuous grinding. Before aging treatment, the total mass loss rate and stage mass loss rate of this abrasive layer after 14h of grinding are 5.79% and 0.15%, respectively. After the aging treatment, the total mass loss rate of the subsequent grinding for 3 h is less than 1%, and the maximum stage mass loss rate does not exceed 0.3%. The specimens fail to show large aggregate spalling after the UV aging treatment.

The most intuitive effect of UV aging on the ultra-thin polyurethane wear layer is the yellowing of the exposed polyurethane material on its surface and the reduction of the bond strength of the binder. However, the reduction in bond strength between the aggregate and the binder cannot break the bond between them. Therefore, the aging wear-resistant layer does not show the phenomenon of accelerated flaking of the aggregate, indicating that the bond strength between the polyurethane, aggregate, and original pavement still meets the requirements for use but is only oxidized due to the absorption of ultraviolet light.

(5) Pulling performance

Table 10 shows that the pull-out strength of the polyurethane ultra-thin wear layer exhibits a decrease of only 2.59% after 16 days (two years outdoors) of UV aging. Combined with the analysis of the infrared spectra of polyurethane above, it can be seen that UV aging makes the molecular bonds inside the polyurethane break, reducing its internal strength. However, the reduction is not large, indicating that the polyurethane wear layer has a good anti-UV aging performance.

## 4. Conclusions

Ultra-thin abrasive polyurethane layers were prepared, and their anti-skid properties and anti-peeling properties were studied through indoor tests. The main conclusions obtained were as follows.

(1) The lowest mass loss rate of 4.97% has been observed for ceramic grains, which have the smoothest surface, while the highest mass loss rate of 5.94% was observed for adamantine, which has the roughest surface. The mass loss rate is 4.52% and 5.79% for aggregates with particle sizes of 1 to 2 mm and 2 to 4 mm, respectively;
(2) A comprehensive prediction model is established, considering the evolution of pavement texture and aggregate spalling. A multivariate quadratic polynomial model of the DFC of the abrasive layer is constructed after introducing the aggregate stage mass loss rate (*σ*) and the total stage mass loss rate (*ω*) as factors, and MPD, λa, and SBI to form the synthesis matrix M1. The coefficient of determination R^2^ of the model is 0.989. The *p*-value of the F-test of the total model is <0.0001, and the *p*-value of the interaction term is 0.0488;
(3) UV aging makes polyurethane materials yellow and destroys their internal C-N and C-O molecular bonds, resulting in damage phenomena such as cracking and etching. After 16 days of accelerated UV aging, the tensile strength and elongation at the break of the specimens decreased by 23.8% and 49.2%, the tensile modulus increased by 51.6%, and the pull-out strength of the wear-resistant layer decreased by 2.59%.

## 5. Future Work

1. Future studies should focus on examining the performances of polyurethane mixtures with different component ratios. By adjusting the ratio of polyurethane to other materials, researchers can better understand how these variations influence the mechanical properties, durability, and skid resistance of the ultra-thin wear layers;
2. It is crucial to further study the interactions between polyurethane and other materials, such as aggregates and bonding agents, at a microscopic and macroscopic level;
3. Additional research is needed to explore the long-term aging effects of polyurethane, particularly when exposed to UV radiation, extreme temperatures, and chemical erosion;
4. Future research should focus on the application of this model in real-world conditions, including its adaptability to different climate zones and traffic intensities.

## Figures and Tables

**Table 1:** The basic performance of polyurethane.

Projects Storage Stability		Results	Test Methods
Storage stability		Quality loss < 1%	Sealed for one month
Coating status		No lumps, crusts, easy to mix	GB/T 3186–2006
Coating appearance		Smooth, white	Visual inspection
Adhesion strength		Level 1	ASTM D3359 [43]
High-temperature stability		260 °C	Thermogravimetric Analyzer TGA-1150
Chemical resistance	Water resistance	No abnormal phenomenon	JTT 712–2008 *
	Acid resistance	No abnormal phenomenon	
	Alkali resistance	No abnormal phenomenon	
	Salt resistance	No abnormal phenomenon	
	Oil resistance	No abnormal phenomenon	

* Note: The title of this standard is ‘pavement anti-skid paint’, which is a Ministry of Communications of the People’s Republic of China industry standard.

**Table 2:** Main technical indicators of aggregates.

Properties	Aggregates				Technical Requirements	Specification
Density/(g/cm^3^)	2.946	2.486	3.122	2.71	-	AASHTO T 84
Water absorption/(%)	1.3	1.6	1.2	0.7	≤2	AASHTO T 84
Hardness/(-)	7.0	6.0	8.0	6.0	≥6	

**Table 3:** Relevant formulas for road surface texture parameters.

Parameter Type	Name	Formula
Range characteristics	Mean profile depth *MPD*	$MPD=\frac{h_{a}+h_{b}}{2}-h$, $h=\frac{1}{n}\sum_{i=1}^{n}h_{i}$
	Contour arithmetic mean deviation *R_a_*	$R_{a}=\frac{1}{n}\sum_{i=1}^{n}\left|h_{i}\right|$
	Contour root mean square deviation *R_q_*	$R_{q}=\sqrt{\frac{1}{n}\sum_{i=1}^{n}h_{i}^{2}}$
	Mean elevation difference *MDE*	$MDE=\frac{\sum_{i=1}^{n-1}DE}{n-1}$
Spacing characteristics	Contour arithmetic mean wavelength *λ_a_*	$\lambda_{a}=2\pi \frac{R_{q}}{\Delta_{a}}$
	Contour root-mean-square wavelength *λ_q_*	$\lambda_{a}=\frac{R_{a}}{\Delta_{q}}$
Shape characteristics	Contour arithmetic mean slope Δ*_a_*	$\Delta_{a}=\frac{1}{n-1}\sum_{i=1}^{n-1}\left|\frac{\Delta h_{i}}{\Delta x_{i}}\right|,\Delta h_{i}=h_{i+1}-h_{i},\Delta_{i}=x_{i+1}-x_{i}$
	The slope of the root mean square of the profile Δ*_q_*	$\Delta_{q}=\sqrt{\frac{1}{n-1}\sum_{i=1}^{n-1}{\left(\frac{\Delta h_{i}}{\Delta x_{i}}\right)}^{2}}$
Comprehensive Features	Skewness *Rsk*	$Rsk={\sum_{i-1}^{n}h_{i}^{3}\left(nR_{q}^{3}\right)}^{-1}$
	Kurtosis *Ku*	$Ku=\sum_{i=1}^{n}h_{i}^{4}{\left(nR_{q}^{4}\right)}^{-1}$
	Surface wear index *SBI*	$SBI=\frac{R_{q}}{H_{5\%}}$

Note: ① *n* is the number of section sampling points; ② *H*_5%_ is the surface height of the 5% abrasion area.

**Table 4:** Determination coefficient of correlation between parameters R^2^.

Indicators	DFC	MPD	Ra	Rq	Δa	Δq	λa	λq	Rsk	Ku	MDE	SBI
DFC	1.000	0.708	0.707	0.762	0.389	0.628	0.124	0.472	0.006	0.047	0.497	0.031
MPD	0.708	1.000	0.857	0.814	0.481	0.807	0.180	0.650	0.081	0.029	0.587	0.044
Ra	0.707	0.857	1.000	0.964	0.577	0.855	0.163	0.637	0.000	0.000	0.656	0.002
Rq	0.762	0.814	0.964	1.000	0.542	0.798	0.142	0.587	0.001	0.002	0.635	0.001
Δa	0.389	0.481	0.577	0.542	1.000	0.834	0.738	0.843	0.004	0.004	0.970	0.047
Δq	0.628	0.807	0.855	0.798	0.834	1.000	0.465	0.890	0.003	0.001	0.885	0.007
λa	0.124	0.180	0.163	0.142	0.738	0.465	1.000	0.688	0.001	0.002	0.682	0.017
λq	0.472	0.650	0.637	0.587	0.843	0.890	0.688	1.000	0.004	0.001	0.882	0.002
Rsk	0.006	0.081	0.000	0.001	0.004	0.003	0.001	0.004	1.000	0.001	0.000	0.547
Ku	0.047	0.029	0.000	0.002	0.004	0.001	0.002	0.001	0.001	1.000	0.004	0.021
MDE	0.497	0.587	0.656	0.635	0.970	0.885	0.682	0.882	0.000	0.004	1.000	0.013
SBI	0.031	0.044	0.002	0.001	0.047	0.007	0.017	0.002	0.547	0.021	0.013	1.000

**Table 6:** DFC regression model factor test results.

Factor	Degree of Freedom	Sum of Squares	Mean Square	F-Value	Pr > F
x1	6	0.000054943	0.000009157	2.93	0.1080
x2	6	0.000015530	0.000002588	0.83	0.5870
x3	6	0.000021082	0.000003514	1.13	0.4446
x4	6	0.000007295	0.000001216	0.39	0.8619
x5	6	0.000019112	0.000003185	1.02	0.4904

**Table 7:** DFC model parameter tests.

Models	Degree of Freedom	Type I Sum of Squares	Decision Factor R^2^	F-Value	Pr > F
Linear term	5	0.000226	0.9403	1105.08	<0.0001
Secondary items	4	0.000009883	0.0416	48.37	<0.0001
Interaction items	10	0.000001677	0.0071	4.10	0.0488
Total Model	19	0.000237	0.9890	290.42	<0.0001

**Table 8:** DFC model factor test.

Factor	Degree of Freedom	Sum of Square	Mean Square	F-Value	Pr > F
MPD	6	0.000001352	0.000000225	5.52	0.0283
λa	6	0.000000972	0.000000162	3.81	0.0409
SBI	6	0.000001321	0.000000220	5.59	0.0275
σ	5	0.000011214	0.000001869	82.30	<0.0001
ω	6	0.000007734	0.000001289	37.40	0.0002

**Table 9:** Shear-strength test results of composite specimens at different temperatures.

Temperature/°C	Average Shear Force/N	Coefficient of Variation/%	Average Shear Strength/MPa
−15	25,343.04	1.16	3.58
25	11,123.26	2.85	1.57
50	4186.24	7.49	0.59

**Table 10:** Comparison of pull-out strength of wear layer before and after aging.

Specimen Condition	Tensile Force 1/N	Tensile Force 2/N	Tensile Force 3/N	Average Tension/N	Tensile Strength/MPa
Before aging	2284.5	2273.9	2247.7	2268.7	1.16
After aging	2120.2	2087.2	2057.5	2088.3	1.13

## Data Availability

The datasets generated during analyzed during the current study are available from the corresponding author on reasonable request.
